# Audit of Cancer Clinical Trials in India

**DOI:** 10.1200/JGO.19.00156

**Published:** 2019-07-08

**Authors:** Arya Mariam Roy, Aju Mathew

**Affiliations:** Arya Mariam Roy, MD, University of Arkansas for Medical Sciences, Fayetteville, AR; and Aju Mathew, MD, MPhil, FACP, University of Kentucky, Lexington, KY; and Welcare Hospital, Kochi, India

## TO THE EDITOR:

In their recent article in *Journal of Global Oncology*, Ramaswami et al^1^ studied disparities in breast, lung, and cervical cancer clinical trials worldwide. They observed that there were fewer clinical trials in low- and middle-income countries and also fewer early-phase trials. We would like to point out that only a subset of trials from low- and middle-income countries are registered in the databases that the authors used for their analysis (ClinicalTrials.gov and the World Health Organization International Clinical Trials Registry Platform). Herein we provide detailed data regarding the cancer clinical trials conducted in India.

We conducted an audit of cancer clinical trials registered in the Clinical Trial Registry of India from July 2007 to May 8, 2017. We found 559 studies during that period. Only 350 of these studies were interventional trials. By disease site, the number of interventional trials conducted in India were as follows: breast cancer, 30% (99 trials); lung cancer, 14.8% (49 trials); head and neck cancer, 14.2% (47 trials); multiple tumor types, including hematologic, 11.5% (39 trials); gynecologic malignancies, 9.7% (32 trials); prostate, 6.1% (20 trials); colon, 5.2% (17 trials); other GI sites, 2.1% (seven trials); hepatobiliary, 1.8% (six trials); gastric and pancreatic studies, 1.5% each (five trials); and renal malignancies, 1.2% (four trials). Most of the trials conducted were phase III (45% [150 trials]), phase II (23.7% [83 trials]), and phase I (5% [18 trials]). Randomized trials accounted for 76.6% of studies.

Of the 350 interventional trials, 68% of trials were conducted only in India, and 32% of trials were conducted in India as part of a multinational study. Pharmaceutical companies sponsored 48.9% of the trials (171 trials). Thirty-two percent of trials were sponsored by the government, and 9% of trials were sponsored by a nongovernmental organization.

The key finding of our audit of cancer clinical trials in India is the likely disproportionate attention to certain disease sites of high cancer burden in India. Although head and neck, GI, and gynecologic cancers are among the most prevalent cancers in India, there are fewer trials conducted in these disease sites.^2^ Policy makers and funders may focus on supporting trials in cancer sites that are of high burden in India.

To our knowledge, our study is the most comprehensive audit of cancer clinical trials in a developing country. Because more nations are launching their own trial registration databases, it is important to conduct routine audits to ensure compliance with the process and to describe the patterns of clinical trials in the region.^3^ Trial registration is a key component of a functional clinical trials infrastructure. Unfortunately, any similar study is limited by the quality of the data input into the registration database. The investigators and sponsors must ensure accuracy of the trial registration in these databases.

Access to clinical trials is limited in low- and middle-income countries. For instance, in India, although the nation accounts for nearly one-fifth of the global population, the proportion of active global clinical trials is less than 2%.^4^ Many factors, such as confusing legal regulations, lack of research infrastructure, and lack of clinician awareness about the importance of clinical research trials, may explain the smaller number of trials in low- and middle-income countries compared with high-income countries. We have to systematically target these barriers to ensure that patients in developing regions of the world have access to clinical trials.
